# Positive Allosteric Modulation of CD11b as a Novel Therapeutic Strategy Against Lung Cancer

**DOI:** 10.3389/fonc.2020.00748

**Published:** 2020-05-21

**Authors:** Terese Geraghty, Anugraha Rajagopalan, Rabail Aslam, Alexander Pohlman, Ishwarya Venkatesh, Andrew Zloza, David Cimbaluk, David G. DeNardo, Vineet Gupta

**Affiliations:** ^1^Department of Internal Medicine, Drug Discovery Center, Rush University Medical Center, Chicago, IL, United States; ^2^Division of Hematology, Oncology and Cell Therapy, Department of Internal Medicine, Rush University Medical Center, Chicago, IL, United States; ^3^Department of Pathology, Rush University Medical Center, Chicago, IL, United States; ^4^Department of Medicine, Washington University School of Medicine, St. Louis, MO, United States; ^5^Siteman Cancer Center, Washington University School of Medicine, St. Louis, MO, United States; ^6^Department of Pathology and Immunology, Washington University School of Medicine, St. Louis, MO, United States

**Keywords:** CD11b, activation, macrophages, immunotherapy, lung cancer

## Abstract

Lung cancer is one of the leading causes of cancer-related deaths in the United States. A major hurdle for improved therapies is immune suppression mediated by the tumor and its microenvironment. The lung tumor microenvironment (TME) contains large numbers of tumor-associated macrophages (TAMs), which suppress the adaptive immune response, increase neo-vascularization of the tumor, and provide pro-tumor factors to promote tumor growth. CD11b is highly expressed on myeloid cells, including TAMs, where it forms a heterodimeric integrin receptor with CD18 (known as CD11b/CD18, Mac-1, CR3, and αMβ2), and plays an important role in recruitment and biological functions of these cells, and is a validated therapeutic target. Here, we describe our pre-clinical studies targeting CD11b in the context of lung cancer, using pharmacologic and genetic approaches that work via positive allosteric modulation of CD11b function. GB1275 is a novel small molecule modulator of CD11b that is currently in Phase 1/2 clinical development. We assess GB1275 treatment effects on tumor growth and immune infiltrates in the murine Lewis Lung Carcinoma (LLC) syngeneic tumor model. Additionally, as an orthogonal approach to determine mechanisms of action, we utilize our recently developed novel CD11b knock-in (KI) mouse that constitutively expresses CD11b containing an activating isoleucine to glycine substitution at residue 332 in the ligand binding CD11b A-domain (I332G) that acts as a positive allosteric modulator of CD11b activity. We report that pharmacologic modulation of CD11b with GB1275 significantly reduces LLC tumor growth. CD11b KI mice similarly show significant reduction in both the size and rate of LLC tumor growth, as compared to WT mice, mimicking our observed treatment effects with GB1275. Tumor profiling revealed a significant reduction in TAM infiltration in GB1275-treated and in CD11b KI mice, increase in the ratio of M1/M2-like TAMs, and concomitant increase in cytotoxic T cells. The profiling also showed a significant decrease in CCL2 levels and a concomitant reduction in Ly6C^hi^ monocytes in circulation in both groups. These findings suggest that positive allosteric modulation of CD11b reduces TAM density and reprograms them to enhance the adaptive immune response and is a novel therapeutic strategy against lung cancer.

## Introduction

Lung cancer is a leading cause of cancer-related deaths worldwide and accounts for more deaths than breast, prostate, and colon cancers combined (1). Conventional treatments against lung cancer are not very effective, partly due to tumor heterogeneity and its complex mutational landscape (2). Recent advances utilizing immune checkpoint inhibitors have made a significant impact on reducing tumor growth and metastases and have resulted in significantly improved survival of lung cancer patients (3, 4). However, these agents show efficacy in only a subset of patients and are associated with significant adverse events (5). Thus, there is an urgent need for improved therapeutics against lung cancer.

Tumor-associated macrophages (TAMs) play a significant role in suppressing the immune response against cancer and alter patient outcomes, depending on their abundance and activity (6–9). TAMs augment cancer immune escape by multiple means, including providing growth factors for tumor survival, increasing neoangiogenesis, suppressing T cell influx and T cell-mediated immunity, and influencing chemokine signaling and extracellular matrix (ECM) integrity to enhance tumor cell extravasation and metastasis (10–15). TAMs represent one of the most abundant leukocyte populations in the tumor microenvironment (TME) and, for most cancer types, are associated with poor survival (16–20). Thus, there are increasing efforts targeting these cells toward developing new therapies against cancer [reviewed in Ruffell and Coussens (7) and Mantovani et al. (8)]. These targeted therapies include both inhibiting recruitment of new TAMs and manipulating the polarization state of resident macrophages.

Although macrophages exist across a continuum of polarization states (9, 21, 22) for simplicity they are often categorized into two major types—M1 and M2 (23, 24). M1 macrophages are considered to be pro-inflammatory and anti-tumor, whereas M2 macrophages are considered to be anti-inflammatory and pro-tumor (9). Indeed, a high abundance of M2 macrophages [for example, CD204^+^ TAMs (25)] in the TME is often associated with poor clinical outcomes in patients, while an increase in M1 macrophage (for example, HLA-DR^+^ TAMs) abundance in tumors often correlates with better prognosis for lung cancer patients (26, 27).

TAMs rely on chemokine cues and cell adhesion molecules for trafficking to the TME, including highly expressed heterodimeric integrin receptors, such as α4β1 and CD11b/CD18 (also known as Mac-1, CR3, and αMβ2) (20, 28–30). The integrin heterodimer CD11b/CD18 is highly expressed on myeloid cells and plays an important role in cell adhesion, migration to inflammatory sites, and phagocytosis (31). It also modulates pro- and anti-inflammatory signaling in cells (32). Thus, CD11b/CD18 is an ideal receptor for targeting TAMs as a means by which tumor growth can be controlled. However, many previous approaches for targeting this family of integrins, especially using antagonists or inhibitors of ligand binding have not been clinically very successful (33). Additionally, we recently found that global deletion of CD11b in mice (CD11b^−/−^) results in increased tumor volume compared to that of wild type (WT) animals and produces a significantly increased tumor burden, suggesting that CD11b plays an important role in controlling tumor growth (34). This also suggests a need for alternate strategies targeting CD11b.

Toward this, we recently developed novel small molecule modulators of CD11b (30, 34, 35). Counter-intuitively, these modulators reduce leukocyte cell migration and tissue recruitment by increasing CD11b-dependent cell adhesion *in vivo* (36). These compounds bind to the ligand-binding domain of CD11b [known as CD11b A- or I-domain (31) CD11bA]. Specifically, they bind an allosteric pocket of CD11bA, thus, positively modulating the domain (and the integrin) into its ligand-competent conformation (35). As such these serve as positive allosteric modulators of CD11b, and ultimately suppress myeloid cell recruitment into inflamed tissues, thereby reducing injury (32, 35). Additionally, we recently showed that the orally bioavailable CD11b positive allosteric modulator, GB1275 [previously known as ADH-503 (30)] significantly reduces growth of pancreatic tumors in multiple mouse models and improves survival (30). However, efficacy of this oral agent has not been described in lung cancer. Here, we present our studies with this agent in a murine lung cancer model. Importantly, in order to complement our pharmacologic approaches of modulating CD11b, we also recently developed a novel CD11b knock-in (KI) mouse that globally incorporates the point mutation, I332G, in the allosteric pocket, which modulates a CD11bA conformational change (34, 37). This mutation allosterically renders CD11bA (and the integrin) ligand competent, in much the same way as GB1275, thereby providing us with a unique tool by which we further validate our mechanism of action in a murine lung cancer model. Together, this study demonstrates a novel approach to treating lung cancer via positive modulation of CD11b.

## Materials and Methods

### Animal Models

Lewis lung carcinoma (LLC) cells were purchased from ATCC. For each experiment, LLC cells were thawed and passaged at least three times before inoculating into mice. Cells were routinely checked and found negative for mycoplasma using the MycoAlert assay (Lonza). Eight to ten week old C57BL/6 wildtype (WT) mice utilized in these experiments were obtained from The Jackson Laboratory. Both male and female mice were used. We have previously described our CD11b (I332G) knock-in (KI) mouse model, backcrossed onto the C57BL/6 background (34). To establish LLC tumors in mice, we subcutaneously inoculated 1.0 × 10^6^ cells in 100 μL cold phosphate-buffered saline (PBS) into the mouse rear flank, for accessible continuous tumor monitoring. Tumors became palpable (>0.5 cm) 5–7 days after inoculation. Mice with palpable tumors were randomized into groups and some mice were treated. Mice were euthanized at the described endpoints or when a tumor measured 2 cm in any diameter (38). The Institutional Animal Care and Use Committee (IACUC) at Rush University Medical Center approved all animal studies.

### Treatments

GB1275 [previously known as ADH-503 (30)] was used at a dose of 60 mg/kg in all tumor studies. A GB1275 stock was prepared in a vehicle solution of 0.5% Methylcellulose (Calbiochem) and 0.02% Tween-80 (Sigma) in water. Control animals were administered the vehicle alone. GB2175 or the vehicle was administered to animals twice daily by oral gavage (o.g.). For T cell depletion, CD4- and CD8-depleting antibodies (anti-mCD4 clone GK1.5 and anti-mCD8 clone 2.43, BioXCell) were administered via intraperitoneal (i.p.) injection every 3–4 days, with the first injection at 500 μg of antibody/animal before tumor implantation and subsequent injections at 250 μg of antibody/animal (39). CCR2i (PF-04136309, Pfizer) was used for the *in vitro* migration assay at 20 nM.

### Mouse Tissue Harvesting and Flow Cytometry

Mice were anesthetized using Ketamine/Xylazine before blood collection was performed via orbital venous puncture. Collected blood was allowed to clot at room temperature for 2 h before centrifugation (at 5,000 RPM for 5 min) to collect sera for analyses. Sera were stored at −80°C until analyses. Tumor tissues were manually dissected, minced, and digested in 10 mL Hank's Balanced Salt Solution (HBSS) containing 0.5 mg/mL Collagenase IV, 0.1 mg/mL Hyaluronidase V, 0.6 U/mL Dispase II, and 100 U/mL DNase I, for 30 min at 37°C with gentle shaking. The digestion was stopped by adding 10 mL PBS containing 0.5% bovine serum albumin (BSA) and 2 mM EDTA. Tumor tissues were then filtered through a 70 μm nylon mesh and subsequently red blood cells (RBC) were lysed by incubating with RBC lysis buffer containing ammonium chloride for 5 min (Biolegend). Single-cell suspensions were stained for flow cytometry analysis with fluorophore-conjugated anti-mouse antibodies at dilutions following manufacturer recommendations. Data were acquired using an LSR-Fortessa flow cytometer and analyzed using FlowJo software version 10.2. Gating strategies were designed according to previously published studies (30, 34). All antibodies used for flow cytometry analysis are listed in Supplementary Table 1.

### Human NSCLC Tissue

Human tissues were obtained from surgically resected specimens from NSCLC patients diagnosed in the Department of Pathology at Rush University Medical Center and accessed via the Rush University Biorepository Core. Patients from whom these tissues were obtained were positive for end-stage disease and did not receive neoadjuvant therapy. Tissues were embedded in paraffin blocks and processed into 7 μm sections for immunohistochemistry (IHC) staining. All tissues were collected under informed consent and access to the biorepository was approved by the Institutional Review Board (IRB) at Rush University Medical Center under study protocol #17060903-IRB02.

The TCGA NSCLC overall survival data (TCGA LUAD) was analyzed using Kaplan–Meier Plotter survival analysis software (http://kmplot.com/analysis/) (40). CD11b (*ITGAM*—probe 205785_at) expression was categorized at the top- and the bottom-third terciles (67th and 33rd percentile) and patient subpopulations were designated as CD11b-high and CD11b-low based on these cutoffs. Survival outcome in CD11b-high vs. CD11b-low patient subpopulations was examined using Kaplan–Meier log-rank test and the univariate Cox proportional hazards regression analysis prebuilt in the K–M plotter. For determining the correlation between CD11b and CCL2 expression in patient samples, the TCGA gene expression data on Lung Adenocarcinoma (LUAD) patients was retrieved via the FireBrowse tool from the Broad Institute. Data was confined to patient diseased tissue, and all normal human tissue samples were removed, leaving 125 patient samples. CD11b and CCL2 expression data were both log2 transformed to assess for fold-change in expression. Excel version 14.7.7 was used to calculate Pearson correlations between these log2-transformed data.

### Histology

Tissues were fixed in 10% formalin overnight while shaking. Tissues were then dehydrated in increasing grades of ethanol and xylene, embedded in paraffin, and cut into 7-μm sections. All IHC on human samples was performed at the University of Illinois at Chicago Histology Core using an BONDRx automated immunostainer (Leica), and slides were scanned using an Aperio AT2 scanner (Leica). A rabbit monoclonal anti-human CD11b antibody (clone EP1345Y, Abcam) was used for IHC staining of CD11b. Tissues were quantified with Aperio ImageScope software (Leica) using an algorithm in HALO software (Indica Labs) to detect positive cells among total cells per tissue, after negative panning of necrotic or tissue-folded areas.

### Bone Marrow-Derived Macrophage Culture

Bone marrow cells were aseptically harvested from 8- to 10-week-old mice, as previously described (41). Bone marrow-derived macrophages (BMDMs) were obtained by differentiating the harvested cells in Dulbecco's Modified Eagle Medium (DMEM) containing 20% FBS and 1% penicillin/streptomycin (PS) and 50 ng/mL M-CSF for 7 days (41). Tumor-conditioned media (TCM) was collected from LLC supernatants after 2 days of growth. After subculture, LLC cells were allowed to grow for 24 h and serum-starved for another 24 h before the supernatant was collected. Serum-starved LLC TCM was centrifuged (1,200 RPM for 5 min) and filtered (0.22 μm) before freezing. After differentiation, BMDMs were stimulated with thawed LLC TCM for 4 h with or without GB1275 at a final concentration of 20 μM.

### qRT-PCR

Total RNA was isolated from cells and tissue using the Qiazol kit (Qiagen), as per manufacturer instructions, and kept frozen at −80°C until extraction. Total mRNA was extracted using the RNeasy kit (Qiagen), according to manufacturer protocols. cDNA synthesis was performed on 1 μg total RNA using the iScript reverse transcriptase kit (Biorad). Quantitative PCR was performed using commercially available mouse primers for *Gapdh, Arg1, Il10, Tgfb, Ccl2, Il1b, Il12b, Cxcl9*, and *Ifna* using Quantitect primer assays (Qiagen). SYBR Green 2X Master Mix was used for qPCR analysis (Biorad). mRNA levels were normalized to *Gapdh* levels (dCt = Ct target gene – Ct *Gapdh*) and reported as relative mRNA expression [2^(−ddCt)^ where ddCt = dCt experimental – dCt control] (Qiagen).

### Migration Assay and ELISA

The THP-1 human monocyte cell line was obtained from ATCC and tested negative for mycoplasma. CCR2i (PF-04136309) was obtained from MedChem Express. Monocytes were pre-treated with GB1275 (20 μM), CCR2i (20 nM), or vehicle control for 30 min prior to seeding (0.5 × 10^6^ cells per well) in the upper chambers of a 24-well transwell plate (Corning, 8 μm). The bottom chambers were filled with media containing either vehicle, CCL2 (50 ng/mL), or TCM. Monocytes that transmigrated to the bottom wells after 6 h were collected and counted using an automated cell counter (ViCellXR). Percent migration was calculated as: (cells migrated in the bottom chamber)/(total cells seeded in the upper chamber) × 100.

LLC tumor tissue was snap frozen in liquid nitrogen and stored at −80°C. Tumor tissue was weighed and lysed by sonication in RIPA lysis buffer containing protease inhibitor (2.5 μL per 1 mg of tissue). Tissue lysis occurred for 30 min on ice before samples were centrifuged (13,000 RPM for 15 min at 4°C). The lysate was collected and protein concentration was determined by BCA assay. CCL2 protein was detected by ELISA (R&D systems) following the manufacturer's protocol.

### Statistics

Statistical analyses were performed using Prism 8.2 software (GraphPad Software). Two-tailed student's *t*-test (two groups) and ANOVA (multiple groups) were utilized when the data were normally distributed. Mann-Whitney test was used for comparison between two groups when data was not normally distributed. Animals were randomly assigned to groups prior to beginning treatment. All experiments were replicated 2–4 times.

## Results

### CD11b^+^ Cells Are Abundant in Human Non-Small Cell Lung Cancer (NSCLC)

**Figure 1:**
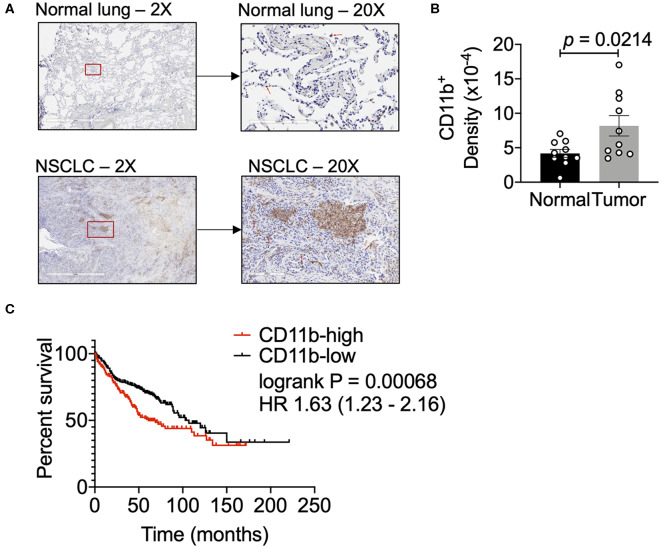
Human lung cancer tissues have a dense CD11b^+^ myeloid population. **(A)** Representative images of human NSCLC and normal lung tissues assessed for CD11b+ cells at 2X and 20X magnifications. Red box in 2X micrographs indicates region magnified at 20X. Red arrows in 20X images indicate CD11b+ cells. Lung tissue sections (7 μM) were stained by IHC using BONDRx automated system (Leica). **(B)** Slides were scanned using Aperio AT2 systems (Leica) and analyzed for total CD11b+ cells over total tissue area (2 μm), reported as CD11b+ density. Slides were negative panned for necrotic or tissue folded areas before quantification. (*n* = 10 per group, *p* = 0.0142). Statistical analysis by Mann-Whitney *t*-test. **(C)** Kaplan–Meier (KM) overall survival (OS) curves of NSCLC patient datasets from TCGA, GEO, and caBIG stratified according to expression of CD11b (*ITGAM*) categorized at the top and bottom thirds, resulting in 242 patients in CD11b-low and 243 patients in CD11b-high group. *P*-value of the log-rank test is shown in the graph. Data presented using K–M plotter (40).

To examine relative abundance of CD11b^+^ myeloid cells in human lung cancer, we compared the density of these cells in human NSCLC vs. normal lung tissue sections by IHC. Clinical characteristics of these samples are described in Supplementary Figure 1. NSCLC tissues showed a significantly higher density of CD11b^+^ leukocytes in the tumor as compared to the normal tissue sections (Figures 1A,B). Additionally, we analyzed the data on lung adenocarcinoma (LUAD) from The Cancer Genome Atlas (TCGA) (42), to determine if CD11b expression in TME correlated with clinical outcomes. Expression levels of CD11b across the LUAD patient groups were categorized at the top and bottom thirds and Kaplan–Meier survival curves were plotted from CD11b-high and CD11b-low groups, with 242 patients in CD11b-low and 243 patients in CD11b-high groups. Our analysis showed that higher CD11b expression is significantly linked with patients with lower survival rate in LUAD (Figure 1C), with median survival of 103 months in the CD11b-low group and 59 months in CD11b-high group. These results are not too surprising, given the recent studies showing presence of a high level of neutrophils and macrophages in the lung TME, providing it with its unique immune contexture (43–45). Collectively, these data suggest that CD11b^+^ cells are increased in the lung TME. Furthermore, CD11b may provide prognostic value and could serve as a potential target for NSCLC therapy.

### Positive CD11b Modulation via Pharmacologic and Genetic Approaches Slows Lung Cancer Tumor Progression

**Figure 2:**
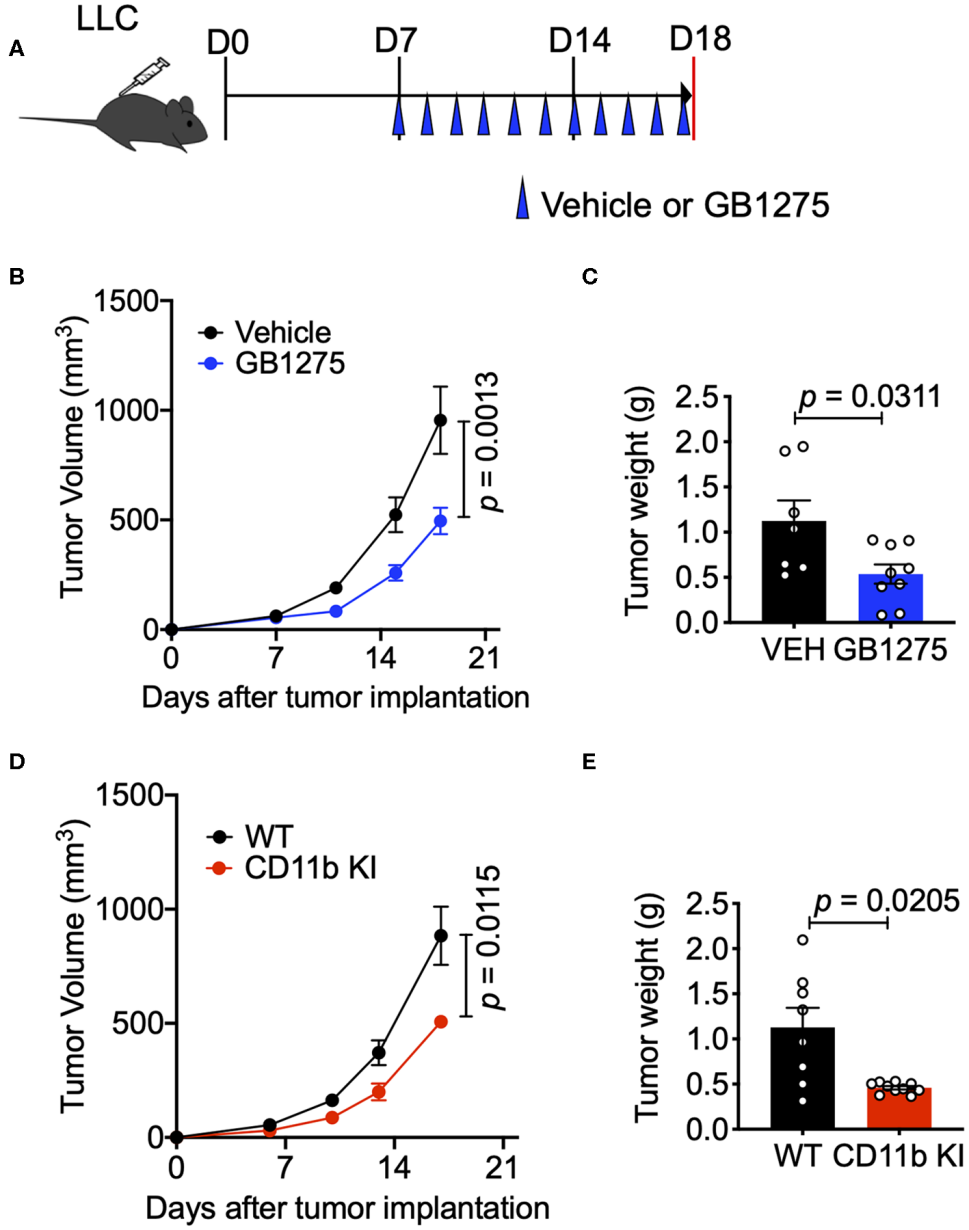
CD11b modulation effectively slows LLC tumor progression and is dependent on T-cell mediated anti-tumor immunity. **(A)** Study design schematic describing the LLC model and treatment regimen of GB1275. **(B)** Wildtype LLC tumor-bearing mice were treated with Vehicle or GB1275. Tumor growth measured as tumor volume are shown (*n* = 7–10 per group). Tumor volume was measured with calipers. **(C)** Endpoint (Day 18) tumor weights are shown from mice treated with Vehicle or GB1275 (*n* = 7–10 per group). **(D)** Transgenic CD11b KI or WT mice were inoculated with LLC and tumor growth was observed over time. Tumor growth measured in tumor volume are shown (*n* = 7–10 per group). **(E)** Endpoint (Day 18) tumor weights are shown from WT or CD11b KI mice (*n* = 7–10 per group). *P*-values as stated on graphs. Statistical analysis by Mann-Whitney *t*-test.

To study whether CD11b modulation affects tumor growth and can be a novel therapeutic approach, we utilized the murine LLC transplantable lung cancer model using syngeneic immunocompetent C57BL/6 mice (38) (Figure 2A). First, we applied a pharmacologic approach by administering CD11b modulator GB1275 to LLC tumor-bearing mice. Our recent studies have shown that global deletion of CD11b (CD11b^−/−^) results in significantly larger tumors in mice, partly due to increase in immunosuppressive TAMs in the TME (34), suggesting that active CD11b may have an important role in controlling tumor growth. Here, we found that treatment of wild type C57BL/6 mice (WT) with GB1275 significantly reduces tumor growth, as compared to animals treated with vehicle alone (Figure 2B). Measurement of tumor weight at the end of the experiment also showed a significant lower tumor weight in GB1275-treated animals as compared to vehicle controls (Figure 2C). These data suggest that pharmacologic modulation of CD11b activity results in significant reduction in lung tumor growth in mice.

Next, to confirm that CD11b modulation was indeed the mechanism by which GB1275 reduced LLC tumor growth, we utilized our newly developed CD11b knock-in (KI) mouse (34) in these experiments. The CD11b KI mouse globally expresses constitutively active CD11b [via a knocked-in I332G substitution (34, 37)], mimicking the CD11b modulation caused by GB1275 treatment. We observed that, upon subcutaneous injection of LLC cells, CD11b KI mice showed a significant reduction in tumor growth, slower tumor growth, and significantly reduced tumor weight at the endpoint as compared to WT mice (Figures 2D,E). Overall, the tumor burden in the CD11b KI resembles the lower tumor burden observed in mice treated with CD11b modulator GB1275. Collectively, these data establish that positive allosteric modulation of CD11b, via orthogonal pharmacologic and genetic approaches reduces growth of lung tumor in mice.

### Positive CD11b Modulation Slows Tumor Progression by Changing Immune Cell Populations Within the TME

**Figure 3:**
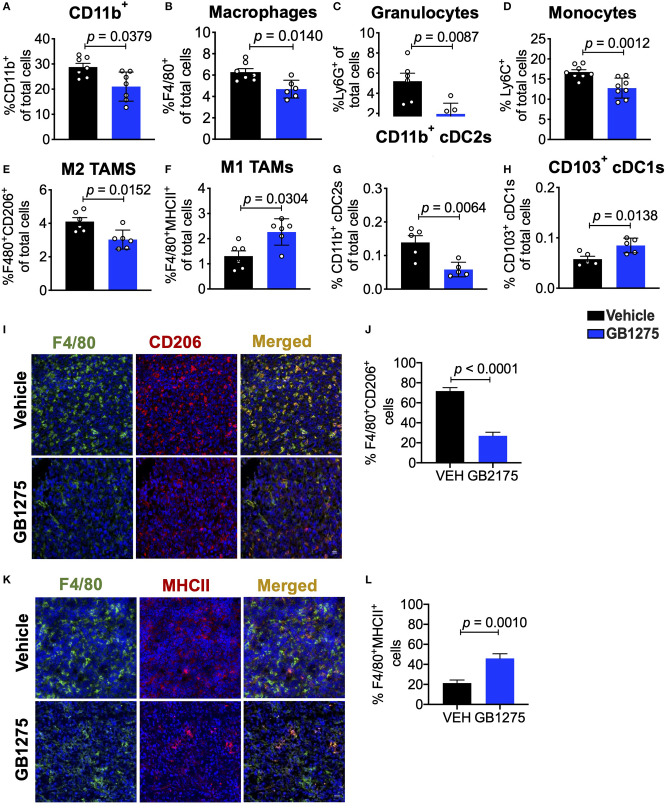
Pharmacologic CD11b modulation alters innate immune responses in the lung tumor microenvironment. **(A)** Quantification of tumor-infiltrating myeloid cells including CD11b+ cells, **(B)** F4/80+ macrophages, **(C)** Ly6G+ granulocytes, **(D)** Ly6C+ monocytes, **(E)** “M1” macrophages (CD11b+F4/80+MHCII+), **(F)** “M2” macrophages (CD11b+F4/80+CD206+), **(G)** CD103+ conventional dendritic cells (cDC1s) and **(H)** CD11b+ cDC2s from LLC tumor tissues treated with Vehicle or GB1275 after 10 days treatment (*n* = 5–10 per group). Populations gated under live single cell CD45+ cells. Gating strategies are shown in Supplementary Figure 1. **(I)** Immunofluorescence (IF) histologic representative images of LLC tumor tissue probing for F4/80 and CD206 in Vehicle or GB1275 treatment groups. **(J)** Quantification IF images in I showing percent colocalization of F4/80+CD206+ macrophages in LLC tumors. **(K)** Same as in panel **(I)**, but showing IF staining on LLC tumor tissues probing for F4/80 and MHCII colocalization. **(L)** Same as in panel **(J)** but, for quantification of IF images in panel **(K)**. *P*-values as stated in graphs. Statistical analysis by Mann-Whitney *t*-test.

Next, we sought to further explore how CD11b modulation slows lung cancer progression. We have previously shown that GB1275 does not reduce viability of tumor or immune cells (34), but rather transmigration and recruitment of CD11b^+^ cells into TME from circulation (32, 35). To fully explore the nature of immune cell changes in the TME, we quantified the various cells of the immune cell compartment in tumors using flow cytometry. We found that GB1275 treatment results in significant reduction in CD11b^+^ cells in isolated tumors, as compared to vehicle-treated mice (Figure 3A). Flow cytometry analyses also showed a significant reduction in F4/80^+^ macrophages (Figure 3B), granulocytes (Figure 3C and Supplementary Figure 1A) and monocytes (Figure 3D and Supplementary Figure 1B) in the TME, further suggesting that pharmacologic modulation of CD11b with GB1275 reduces transmigration and recruitment of CD11b^+^ cells into the lung TME. To further evaluate the effect of pharmacologic CD11b modulation on recruitment of myeloid cells into tumors, we conducted an *in vitro* monocyte migration assay using LLC tumor conditioned media (TCM) (Supplementary Figure 2A). We observed that control myeloid cell chemoattractant CCL2 as well as LLC TCM both elicit strong transmigration of human THP-1 monocytes. GB1275 significantly inhibits monocyte transmigration, comparable in efficacy to CCR2 inhibition (CCR2i) (Supplementary Figure 2B). These results confirm that *in vivo* effects of GB1275 are likely partly mediated via an effect on transmigration of CD11b^+^ cells into the TME. M2 polarized TAMs are associated with lung tumor growth, whereas M1 macrophages reduce tumor growth (25, 26). To examine if GB1275 treatment changes the level of these macrophage sub-types, we utilized polarization-specific markers in flow cytometry, and found that GB1275 significantly increases the frequency of M1 macrophages (F4/80^+^MHCII^+^) and significantly decreases the frequency of M2 macrophages (F4/80^+^CD206^+^) within the TME (Figures 3E,F). Moreover, we found that GB1275 significantly decreases type-2 conventional CD11b^+^ dendritic cells (cDC2) and significantly increases type-1 CD103^+^ dendritic cells (cDC1), suggesting an increase in antigen cross-presentation to CD8^+^ T cells after CD11b modulation (Figures 3G,H and Supplementary Figure 1C), suggesting that the *in vivo* effects of GB1275 on lung cancer are partly also mediated via increasing the ratio of M1/M2 polarized macrophages in the TME, confirming similar findings in pancreatic cancer models (30). To further validate these findings, we analyzed tumors using immunofluorescence (IF), which similarly showed a significant decrease in F4/80^+^CD206^+^ M2-type macrophages (Figures 3I,J) as well as a significant increase in F4/80^+^MHCII^+^ M1-type macrophages (Figures 3K,L) in LLC tumors treated with GB1275 as compared to the vehicle controls.

**Figure 4:**
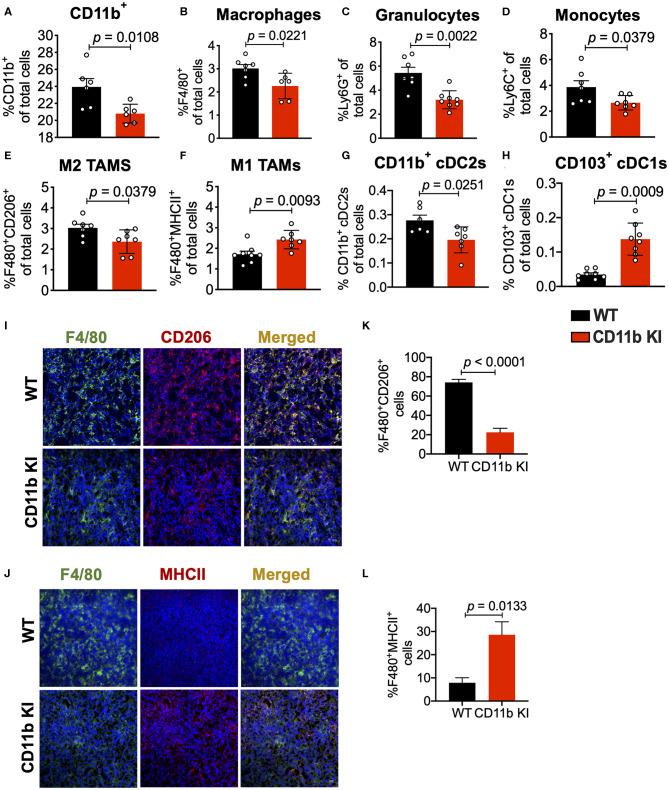
Genetic CD11b modulation alters innate immune responses in the lung tumor microenvironment. **(A)** Quantification of tumor-infiltrating myeloid cells including CD11b+ cells, **(B)** F4/80+ macrophages, **(C)** Ly6G+ granulocytes, **(D)** Ly6C+ monocytes, **(E)** “M1” macrophages (CD11b+F4/80+MHCII+), **(F)** “M2” macrophages (CD11b+F4/80+CD206+), **(G)** CD103+ conventional dendritic cells (cDC1s), and **(H)** CD11b+ cDC2s from LLC tumor tissues in WT or CD11b KI animals 10 days treatment (*n* = 5–10 per group). Populations gated under live single cell CD45+ cells. Gating strategies are shown in Supplementary Figure 1. **(I)** Immunofluorescence (IF) histologic representative images of LLC tumor tissue probing for F4/80 and CD206 in WT or CD11b KI groups. **(J)** Quantification IF images in panel **(I)** showing percent colocalization of F4/80+CD206+ macrophages in LLC tumors. **(K)** Same as in panel **(I)**, but showing IF staining on LLC tumor tissues probing for F4/80 and MHCII colocalization. **(L)** Same as in panel **(J)** but, for quantification of IF images in panel **(K)**. *P*-values as stated in graphs. Statistical analysis by Mann-Whitney *t*-test.

To determine if this mechanism is cellular independent of the way CD11b is functionally modulated, we also analyzed isolated tumors from the LLC tumor bearing CD11b KI mice. Flow cytometry based analyses of the tumors replicated the findings from above (Figures 4A–H) and showed that, as compared to the LLC tumors from WT mice, tumors from the KI mice show significant decrease in CD11b^+^ cells, F4/80^+^ cells, monocytes, and granulocytes, and M2 macrophages, and a significant increase in M1 macrophages. The results also show a significant decrease in CD11b^+^ cDC2s and a significant increase in CD103^+^ cDC1s, further confirming that positive modulation of CD11b is highly effective in controlling lung tumor growth *in vivo*. Furthermore, immunofluorescence-based analyses of the tumor tissues also showed a significant decrease in F4/80^+^CD206^+^ M2-polarized macrophages (Figures 4I,J) as well as a significant increase in F4/80^+^MHCII^+^ M1-polarized macrophages (Figures 4K,L) in LLC tumors from CD11b KI mice, as compared to tumors from the WT animals.

**Figure 5:**
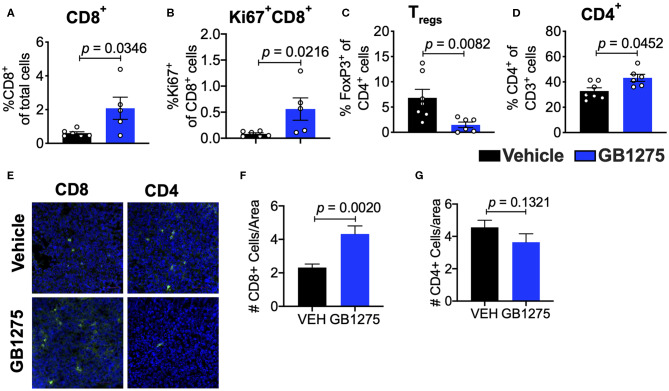
Pharmacologic CD11b modulation alters adaptive immune responses in the lung tumor microenvironment. **(A)** Quantification of tumor-infiltrating T cells including CD8+ cytotoxic lymphocytes (CTLs), **(B)** Ki67+ CD8+ T cells, **(C)** FoxP3+ regulatory T cells (Tregs), and **(D)** CD4+ T cells from LLC tumor tissues treated with Vehicle or GB1275 after 10 days of treatment (*n* = 5–10 per group). **(E)** Immunofluorescence (IF) histologic representative images of LLC tumor tissue probing for CD8 or CD4 in Vehicle or GB1275 treatment groups. **(F,G)** Quantification of CD8 and CD4 IF images in panel **(E)**, respectively. Statistical analysis by Mann-Whitney *t*-test.

**Figure 6:**
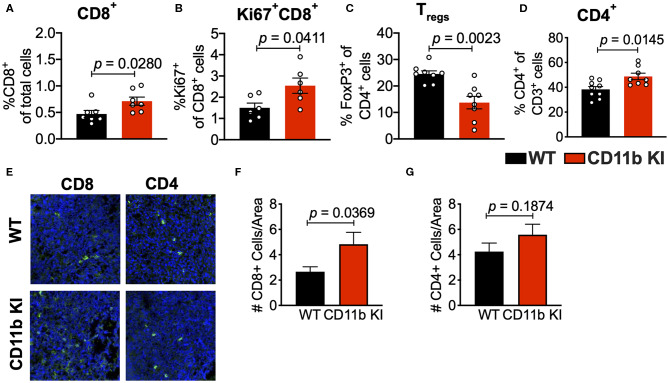
Genetic CD11b modulation alters adaptive immune responses in the lung tumor microenvironment. **(A)** Quantification of tumor-infiltrating T cells including CD8+ cytotoxic lymphocytes (CTLs), **(B)** Ki67+ CD8+ T cells, **(C)** FoxP3+ regulatory T cells (Tregs), and **(D)** CD4+ T cells from LLC tumor tissues treated with Vehicle or GB1275 after 10 days of treatment (*n* = 5–10 per group). **(E)** Immunofluorescence (IF) histologic representative images of LLC tumor tissue probing for CD8 or CD4 in WT or CD11b KI groups. **(F,G)** Quantification of CD8 and CD4 IF images in panel **(E)**, respectively. Statistical analysis by Mann-Whitney *t*-test.

Analyses of the T cell compartment in GB1275-treated tumors showed a coincident increase in the frequency of Ki67+ (proliferating) CD8^+^ T lymphocytes in the TME. GB1275 treatment also led to an increase in CD4^+^ T cells and a concomitant decrease in CD4^+^FoxP3^+^ regulatory T cells (T_regs_) (Figures 5A–D and Supplementary Figures 1D,E). IF staining further confirmed a significant increase in number of CD8^+^ T lymphocytes in GB1275-treated tumors as compared to vehicle controls. IF showed no significant difference between CD4^+^ cells between the two groups (Figures 5E–G). We also similarly analyzed the tumors from CD11b WT and KI mice and found a similar pattern of changes in T cell populations by flow cytometry (Figures 6A–D) and IF (Figures 6E–G).

**Figure 7:**
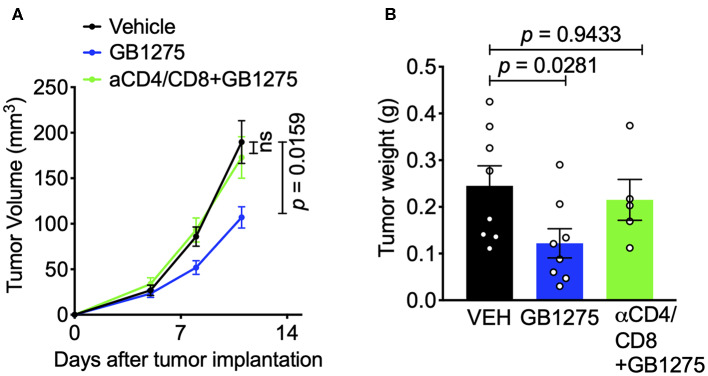
The effects of pharmacologic CD11b modulation are T cell-dependent. **(A)** T cells were depleted from mice using anti-CD4 and anti-CD8 depleting antibodies (BioXCell) and treated GB1275. Tumor growth measured in tumor volume are shown (*n* = 7–10 per group). Tumor volume was measured with calipers. **(B)** Endpoint (Day 12) tumor weights are shown from groups in F (*n* = 5–8 per group). *P*-values as stated on graphs. Statistical analysis by Mann-Whitney *t*-test.

These results suggest that CD11b modulation via pharmacologic and genetic approaches similarly changes the immune phenotype within the TME to augment anti-tumor immune responses and suggests that T cell mediated immunity may be partly responsible for controlling tumor growth. To confirm the role of T cells in controlling tumor growth in this model, we depleted CD4^+^ and CD8^+^ T cells in tumor-bearing mice using an established protocol (39) (Supplementary Figures 3A,B). We found that, in this context, removal of T cells results in loss of GB1275 efficacy (Figures 7A,B). Taken together, these results suggest that CD11b modulation elicits its efficacy in controlling lung tumor growth partly by reducing TAM frequency in tumors and partly by increasing the ratio of M1/M2 macrophages, that results in induction of T cell-mediated anti-tumor immunity.

### Positive Modulation of CD11b Polarizes Macrophages Toward an M1 Phenotype

**Figure 8:**
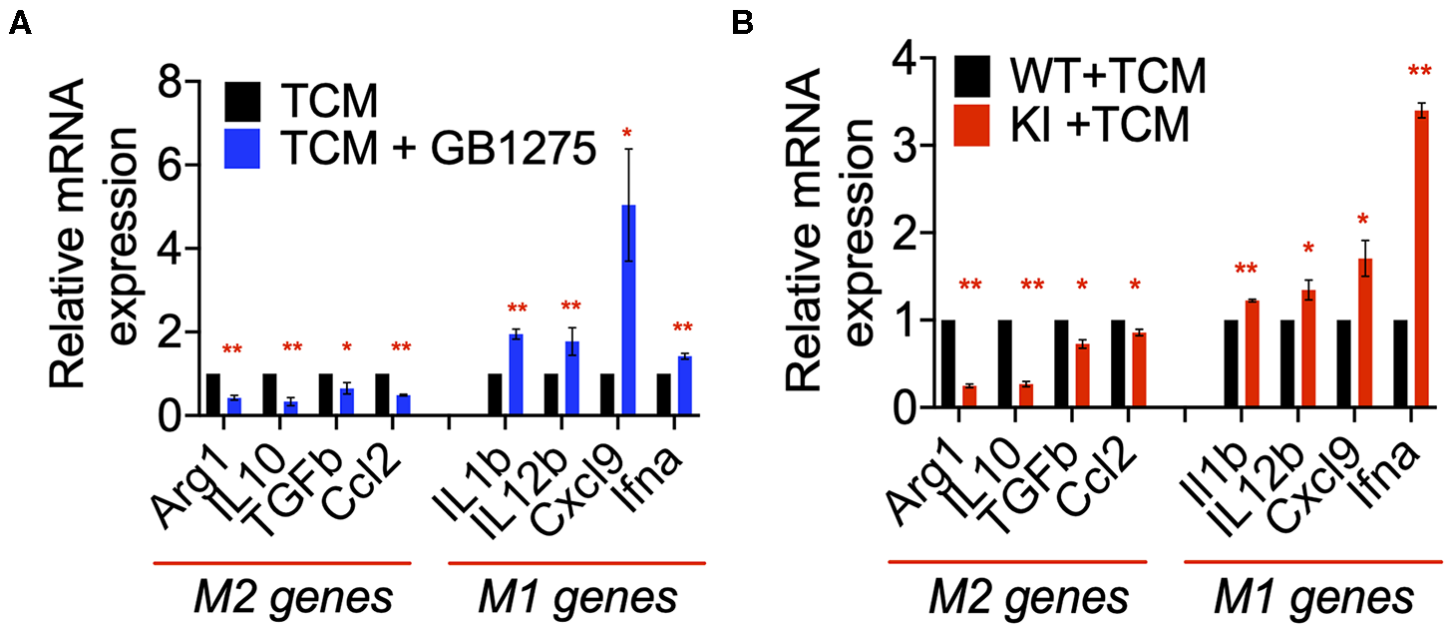
CD11b modulation changes macrophage gene expression to enhance anti-tumor immunity. **(A)** Relative mRNA expression analysis of BMDMs treated with LLC TCM or TCM+GB1275 (20 μM) for 4 h in media containing M-CSF. Changes in gene expression are reported as relative mRNA expression from TCM (*n* = 3–6). **(B)** Q-PCR mRNA expression analysis of BMDMs from WT or CD11b KI mice treated with LLC TCM for 4 h in media containing M-CSF. Changes in gene expression are reported as relative mRNA expression from WT TCM (*n* = 3–6). *P < 0.05, **P < 0.01. Statistical analysis by student's *t*-test.

Our *in vivo* results suggested a role for CD11b-dependent macrophage polarization. Thus, to further examine the changes in macrophage polarization in TME as a result of CD11b modulation, we stimulated primary bone marrow derived macrophages (BMDMs) with LLC tumor-conditioned media (TCM) in the presence or absence of GB1275. We found significant changes in expression level of genes involved in classical (M1) and alternative (M2) activation of macrophages in as a result of CD11b-modulation in the presence of LLC TCM. When stimulated with TCM, GB1275-treated macrophages showed significantly lower expression of M2 genes, including *Arg1, Il10, Tgfb*, and *Ccl2* compared to TCM treatment alone (Figure 8A). Simultaneously, GB1275-treated macrophages showed an increased level of M1 genes, including *Il1b, Il12b, Cxcl9*, and, *Ifna* compared to TCM treatment alone. TCM stimulation of CD11b KI BMDMs also showed similar gene expression pattern, as compared to WT cells (Figure 8B), confirming that CD11b modulation results in increased M1-polarized macrophages in the presence of LLC TCM.

### Positive CD11b Modulation Reduces CCL2-Mediated Monocyte Recruitment

**Figure 9:**
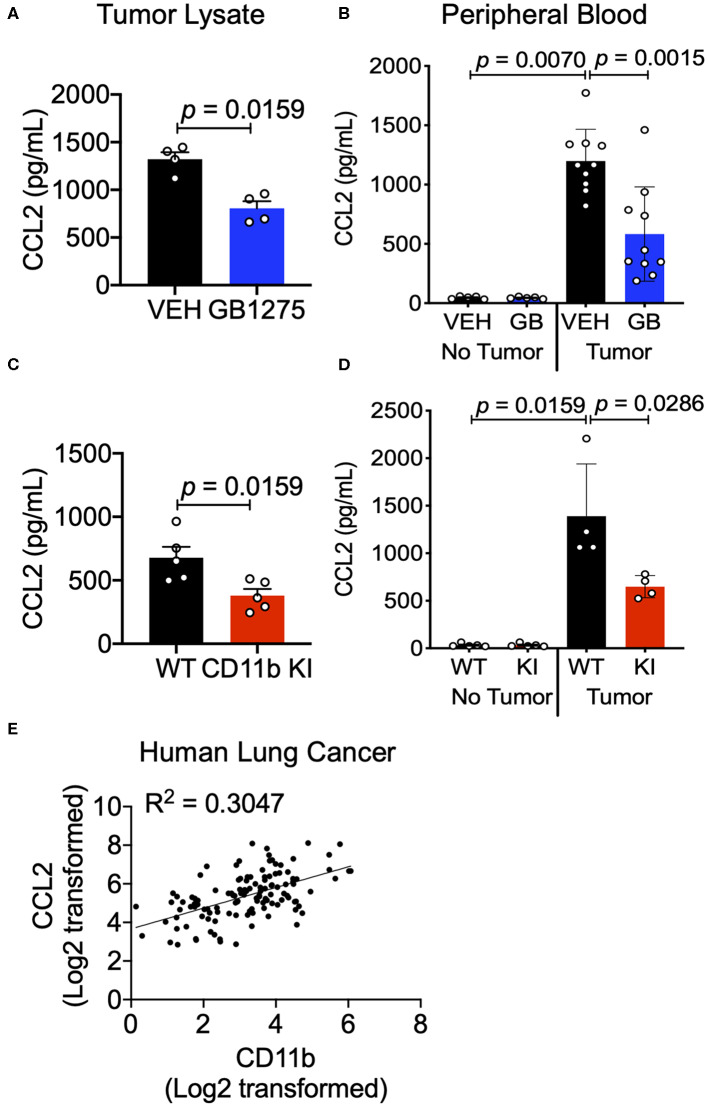
CD11b modulation reduces tumor secreted and circulating CCL2 levels in tumor-bearing mice. **(A)** Levels of CCL2 protein concentration from tumor tissue lysates of healthy controls (no tumor) or endpoint Control (C)- or GB1275 (GB)-treated mice (tumor-bearing) measured by ELISA (*n* = 4–5). **(B)** Levels of CCL2 measured from peripheral blood of non-tumor bearing (No Tumor), or tumor bearing (Tumor) animals (*n* = 4–8) in Vehicle or GB1275 treated animals. **(C)** Same as in panel **(A)**, but from healthy controls (no tumor) or endpoint WT or CD11b KI mice (tumor-bearing) measured by ELISA (*n* = 4–5). **(D)** Same as in panel **(B)**, but in WT or CD11b KI animals. *P*-values as stated in the graphs. Statistical analysis by Mann-Whitney *t*-test. **(E)** Pearson correlation analysis comparing the fold change in CD11b expression levels to the fold change in CCL2 expression levels in Lung Adenocarcinoma samples.

The chemokine CCL2 recruits myeloid cells to the TME *in vivo* and is primarily secreted by lung cancer cells and myeloid cells in the TME (46). Increased CCL2 levels result in a feed forward loop, leading to increased CCR2-mediated TAM cell recruitment (36, 47). Circulating levels of CCL2 also increase in lung cancer patients and correlate with poor survival (48). Neutralizing CCL2, or its receptor CCR2, reduces tumor growth and metastases (47, 48). We examined CCL2 levels in sera of control mice using ELISA and found that they were significantly increased in LLC tumor-bearing animals compared to non-tumor-bearing animals, confirming published reports (36, 46, 47). Analysis of tumor tissue lysates as well as sera from LLC tumor-bearing mice treated with GB1275 showed a significant reduction in circulating CCL2 levels, compared to vehicle-treated controls (Figures 9A,B). Similarly, tumor tissue lysates and sera from LLC tumor-bearing KI mice also showed a significant reduction in circulating CCL2 levels, compared to WT controls (Figures 9C,D). Finally, we observed a strong correlation between CCL2 and CD11b expression from the human TCGA lung adenocarcinoma dataset (Figure 9E). These data suggest that reduced TAM recruitment in the TME, upon pharmacologic and genetic allosteric modulation of CD11b, also results in reduced CCL2 secretion, likely leading to a blocked feed-forward loop in myeloid cell recruitment to the TME.

**Figure 10:**
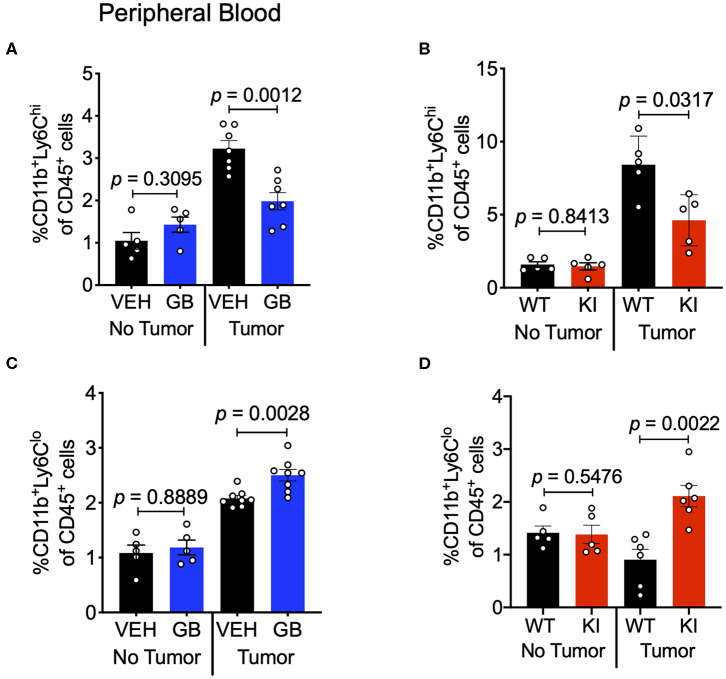
CD11b modulation reduces Ly6C^hi^ inflammatory monocytes in circulation and increases Ly6C^lo^ patrolling monocytes. **(A)** Frequencies of CD11b^+^Ly6G^−^Ly6C^hi^ circulating monocytes of CD45^+^ cells in Vehicle or GB1275 treated mice (*n* = 8) either from non-tumor bearing (No Tumor) or tumor-bearing (Tumor) mouse peripheral blood. **(B)** Same as in panel **(A)**, but showing frequencies for WT vs. CD11b KI animals (*n* = 5). **(C,D)** Same as in panel **(A,B)** respectively, but showing CD11b^+^Ly6G^−^Ly6C^lo^ monocyte frequencies in peripheral blood (*n* = 5–8).

CCL2 in circulation recruits Ly6C^hi^ monocytes from the bone marrow and into the TME, where these cells differentiate into TAMs and typically play a pro-tumoral role (43). Since we observed reduction in CCL2 levels in both the tumor and sera of CD11b-modulated tumors (i.e., GB1275 treated and CD11b KI), we hypothesized that reduced CCL2 in the blood may result in reduced levels of circulating Ly6C^hi^ monocytes. Peripheral blood from vehicle-treated or GB1275-treated tumor-bearing mice was collected and stained for circulating monocytes. In line with our hypothesis, there was a significant decrease in circulating CD11b^+^Ly6G^−^Ly6C^hi^ classical monocytes in both GB1275-treated and CD11b KI tumor bearing mice (Figures 10A,B). Concomitantly, there was a significant increase in Ly6C^lo^ (CD11b^+^Ly6G^−^Ly6C^lo^) monocytes in circulation in both the GB1275-treated and CD11b KI tumor-bearing mice (Figures 10C,D). Increased Ly6C^lo^ and decreased Ly6C^hi^ circulating monocytes suggest more patrolling of endothelium and less systemic inflammatory burden induced by CD11b modulation, which is likely also a contributing factor in how positive modulation of CD11b results in reduced tumor growth *in vivo*.

**Figure 11:**
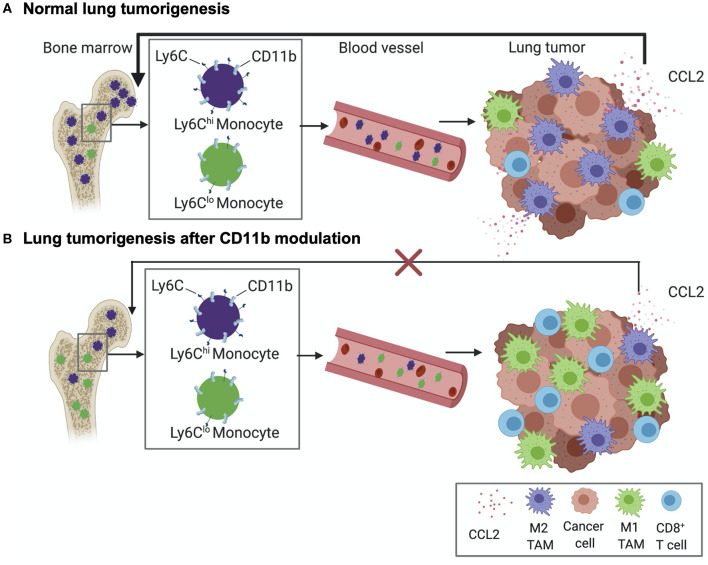
Schematic diagram showing our current working model. **(A)** Normal lung tumor growth secretes high levels of CCL2 to attract CD11b^+^Ly6C^hi^ monocytes into circulation, wherefrom these cells are increasingly recruited to the TME and converted into M2 tumor associated macrophages (TAMs). As a result, a feed-forward loop is established that results in suppression of CD8+ T cells in the TME and promoting lung tumorigenesis. **(B)** Positive allosteric modulation of CD11b reduces recruitment of CD11b^+^Ly6C^hi^ monocytes and, thus, M2-polarized TAM density, reduces CCL2 levels, resulting in suppression of the feed-forward loop. This leads to increased density of tumor-suppressing CD8+ T cells in the TME and suppression of lung tumor growth.

Overall, the above data suggests the following working model of how modulation of CD11b activity regulates tumor growth (Figure 11); Lung tumors, via upregulation of CCL2, recruit Ly6C^hi^CD11b^+^ myeloid cells, which get polarized to an M2- like state in the TME, resulting in increased M2/M1 TAM ratio in the TME in a feed-forward loop. As a result, there is increased T cell immune suppression in the TME, promoting lung tumor growth. However, positive allosteric modulation of CD11b activity suppresses this feed forward loop by decreasing CCL2 levels, reducing Ly6C^hi^CD11b^+^ myeloid cells in circulation and reduced recruitment of these myeloid cells in TME (and thus reduced M2 TAM density in the TME), which leads to increased M1/M2 TAM ratio, increased CD8^+^ T cell infiltration, ultimately resulting in significantly suppressed lung tumor growth. Future studies will probe these mechanisms further.

## Discussion

Targeting tumor-associated myeloid cells has the potential to overcome the limitations of existing immunotherapies for cancer patients (30, 49–53). Increased TAM density in tumors, especially CD204^+^ M2-like TAMs (25), correlates with poor patient prognosis in multiple cancer types, including lung cancer, while HLA-DR^+^ M1-like TAMs correlate with better prognosis for patients (26). These results suggest that an approach that involves a combination of decreasing TAM density and reducing the ratio of M2/M1-like TAMs in TME may be an exciting new therapeutic strategy to treat lung cancer. Our results presented here offer one such approach. Integrin CD11b is highly expressed on TAMs. We determined that CD11b^+^ cells are abundant in human NSCLC samples. Allosteric activation of CD11b using two orthogonal approaches, pharmacologic (using GB1275) and genetic (using a I332G CD11b knock-in), provides significant reductions in the recruitment of F4/80^+^ TAMs in the TME and, concomitantly, results in significant decrease in tumor burden in LLC murine models, which is consistent with our previous results in other model systems (32, 34, 35). Furthermore, we observed an accompanying increase in Ki67^+^CD8^+^ T cells and decrease in FoxP3^+^ regulatory T cells in the TME. These data indicated that CD11b-dependent modulation of TAMs resulted in an increased adaptive immune response, which might be the effectors for controlling tumor growth. Indeed, depletion of all T-cells in GB1275-treated LLC tumor bearing mice showed tumor growth similar to the vehicle treated animals, confirming that T cells are the effector cells controlling tumor growth in this model system. Additionally, we found increased CD103^+^ dendritic cells in the treated animals, which suggests enhanced antigen cross-presentation, likely further enhancing T cell-mediated anti-tumor immunity (15). Collectively, our data highlight positive allosteric modulation of CD11b as a novel strategy for targeting TAM cell recruitment and polarization in TME to enhance both the innate and adaptive immune responses against lung cancer.

Additionally, our findings that CD11b modulation suppresses circulating CCL2 levels in tumor-bearing mice may be an important contributing mechanism defining how CD11b modulation slows tumor growth. Several studies have reported tumor-promoting properties of CCL2 in accelerating metastasis, increasing angiogenesis, and recruiting immune suppressive macrophages to the tumor (47, 54–56). Indeed, lung adenocarcinoma patients with higher levels of CCL2 progress faster and have worse prognosis (48). Additionally, blocking CCL2, or its receptor CCR2, reduces tumor growth and metastases, and this mechanism is currently being target in the clinic (47, 48). Data presented here provides a novel approach to limit CCL2/CCR2 mechanism in solid tumors, perhaps circumventing some of the limitations with the CCL2/CCR2 targeting therapeutics currently under development (55). Future work investigating the link between CD11b modulation and CCL2 signaling will further inform clinical translation of these studies.

In summary, the data presented here show that integrin CD11b is a clinically relevant target in lung cancer, with high abundance seen in NSCLC patient tumors and high CD11b expression correlated with worse overall survival for lung cancer. Our data also show that positive allosteric modulation of CD11b alters both the myeloid cell infiltration and TAM phenotype and functions within the TME to decrease lung cancer burden *in vivo*. A combination of pharmacologic and genetic approaches suggests that these *in vivo* effects are a direct result of CD11b modulation. Thus, positive allosteric modulation of CD11b augments the anti-tumor immune response and is a novel therapeutic strategy against lung cancer.

## Data Availability Statement

The datasets generated for this study are available on request to the corresponding author.

## Ethics Statement

The studies involving human participants were reviewed and approved by The Institutional Review Board at Rush University Medical Center. The patients/participants provided their written informed consent to participate in this study. The animal study was reviewed and approved by The Institutional Animal Care and Use Committee at Rush University Medical Center.

## Author Contributions

TG designed and performed *in vitro* and *in vivo* experiments including mouse model experiments, flow cytometry analysis, and transmigration. DD, AR, IV, and RA assisted in mouse model experiments and flow cytometry analysis. AP and DC helped with histology and imaging analysis. AZ helped with data analysis. TG performed statistical analysis and co-wrote the manuscript. All co-authors edited the manuscript. VG managed and oversaw the project.

## Conflict of Interest

VG is an inventor on patents and applications related to these studies that have been licensed to a company he co-founded (Adhaere Pharmaceuticals, Inc., now part of Gossamer Bio, Inc.). VG has significant financial interest in it. DD is a scientific advisory board member of Adhaere Pharmaceuticals, Inc. The remaining authors declare that the research was conducted in the absence of any commercial or financial relationships that could be construed as a potential conflict of interest. The handling Editor declared a past co-authorship with one of the authors AZ.

## Supplementary Material

Supplementary Figure 1 Representative gating strategies for flow cytometry analysis of tumors. **(A)** Flow diagram representing the beginning gating strategy for all tumor cell flow cytometry analysis. All cells were first gated into single cells using side scatter height by area and then side scatter width by area. Under single cells, live CD45+ cells were gated. Under this gating strategy all subsequent immune cell populations were analyzed. **(B)** Myeloid cell gating strategy starting with gating strategy in panel **(A)**, followed by CD11b+ –> Ly6G –> Ly6C –> F4/80+, and then M1 and M2 macrophages were analyzed as F4/80+MHCII+ and F4/80+CD206+, respectively. Ly6G+ cells are granulocytes and Ly6C+ cells are monocytes. Isotype controls for F4/80, CD206, and MHCII were used to inform gate drawing. **(C)** Dendritic cell (DC) gating strategy, starting with flow in panel **(A)**, followed by Ly6C –> CD11c+MHCII+ –> CD24+F4/80–, and further followed by gating of CD11b+ cDCs or CD103+ DCs. **(D)** CD4 T cell gating, starting with flow in panel **(A)**, followed by CD3+ –> CD4+ –> FoxP3+ cells. Isotype control for FoxP3 was used to inform gate drawing. **(E)** CD8 T cell gating, starting with flow in panel **(A)**, followed by CD3+ –> CD8+ –> Ki67+ cells. Isotype control was used for Ki67 to inform gate drawing. Flow diagrams are representative of gating strategy used in all stained tumor samples.

Supplementary Figure 2 **(A)** Schematic diagram describing the monocyte migration assay. **(B)** Migration assay analysis of THP1 human monocytes treated with media (–), 20 nM CCR2i (PF-4136309) or 20 μM GB1275. Pre-treated or untreated THP1 monocytes (5 × 105) were seeded in the upper chamber and then submerged in media (–), 50 ng/mL CCL2, or tumor conditioned media (TCM) reported as % migration = cells counted in bottom chamber out of total cells seeded in upper chamber. ^*^*P* < 0.05. Statistical analysis by Mann-Whitney *t*-test.

Supplementary Figure 3 Successful *in vivo* depletion of CD4 and CD8 T cell in LLC tumor-bearing mice. **(A)** Representative flow cytometry analysis of tumor cells from vehicle or anti-CD4/CD8 antibody depleted mice. **(B)** Representative flow cytometry analysis of peripheral blood cells from vehicle or anti-CD4/CD8 antibody depleted mice. Gated under live single cell, CD45+CD3+ cells.

Supplementary Table 1 Clinical characteristics of human lung adenocarcinoma tissues stained for CD11b in Figures 1A,B. Normal tissue acquiesced from the Northwestern University biorepository was either cadaveric or designated “non-tumor” normal tissue by pathology report.

Supplementary Table 2 List of antibodies used for flow cytometry analysis.
