# Supplementary figures and images for: Positive Allosteric Modulation of CD11b as a Novel Therapeutic Strategy Against Lung Cancer
Source: Front Oncol. 2020 May 21;10:748. doi: 10.3389/fonc.2020.00748 (PMC7253726; doi:10.3389/fonc.2020.00748)

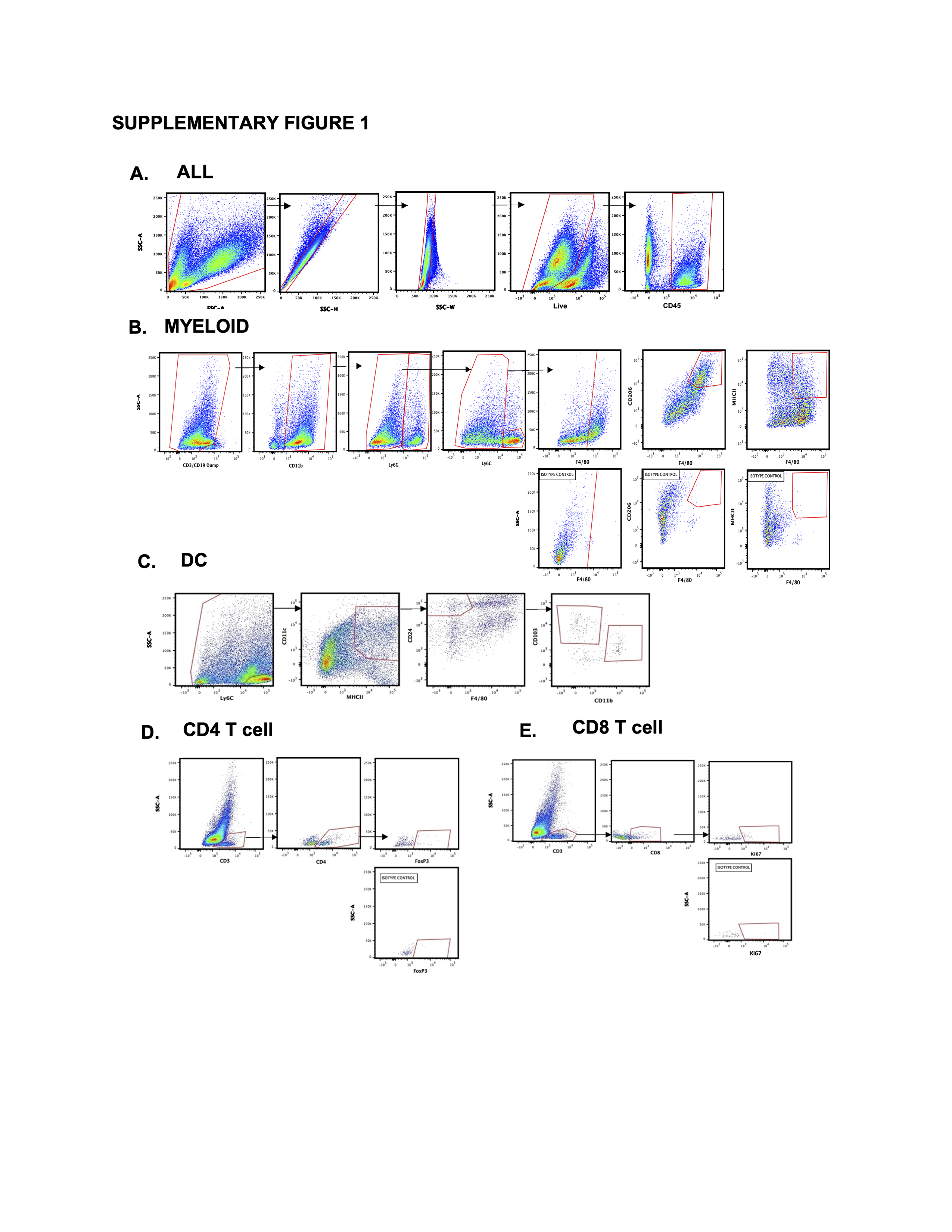

Supplement: Supplementary Figure 1 — Representative gating strategies for flow cytometry analysis of tumors. (A) Flow diagram representing the beginning gating strategy for all tumor cell flow cytometry analysis. All cells were first gated into single cells using side scatter height by area and then side scatter width by area. Under single cells, live CD45+ cells were gated. Under this gating strategy all subsequent immune cell populations were analyzed. (B) Myeloid cell gating strategy starting with gating strategy in panel (A), followed by CD11b+ –> Ly6G –> Ly6C –> F4/80+, and then M1 and M2 macrophages were analyzed as F4/80+MHCII+ and F4/80+CD206+, respectively. Ly6G+ cells are granulocytes and Ly6C+ cells are monocytes. Isotype controls for F4/80, CD206, and MHCII were used to inform gate drawing. (C) Dendritic cell (DC) gating strategy, starting with flow in panel (A), followed by Ly6C –> CD11c+MHCII+ –> CD24+F4/80–, and further followed by gating of CD11b+ cDCs or CD103+ DCs. (D) CD4 T cell gating, starting with flow in panel (A), followed by CD3+ –> CD4+ –> FoxP3+ cells. Isotype control for FoxP3 was used to inform gate drawing. (E) CD8 T cell gating, starting with flow in panel (A), followed by CD3+ –> CD8+ –> Ki67+ cells. Isotype control was used for Ki67 to inform gate drawing. Flow diagrams are representative of gating strategy used in all stained tumor samples. [file Image_1.TIFF]

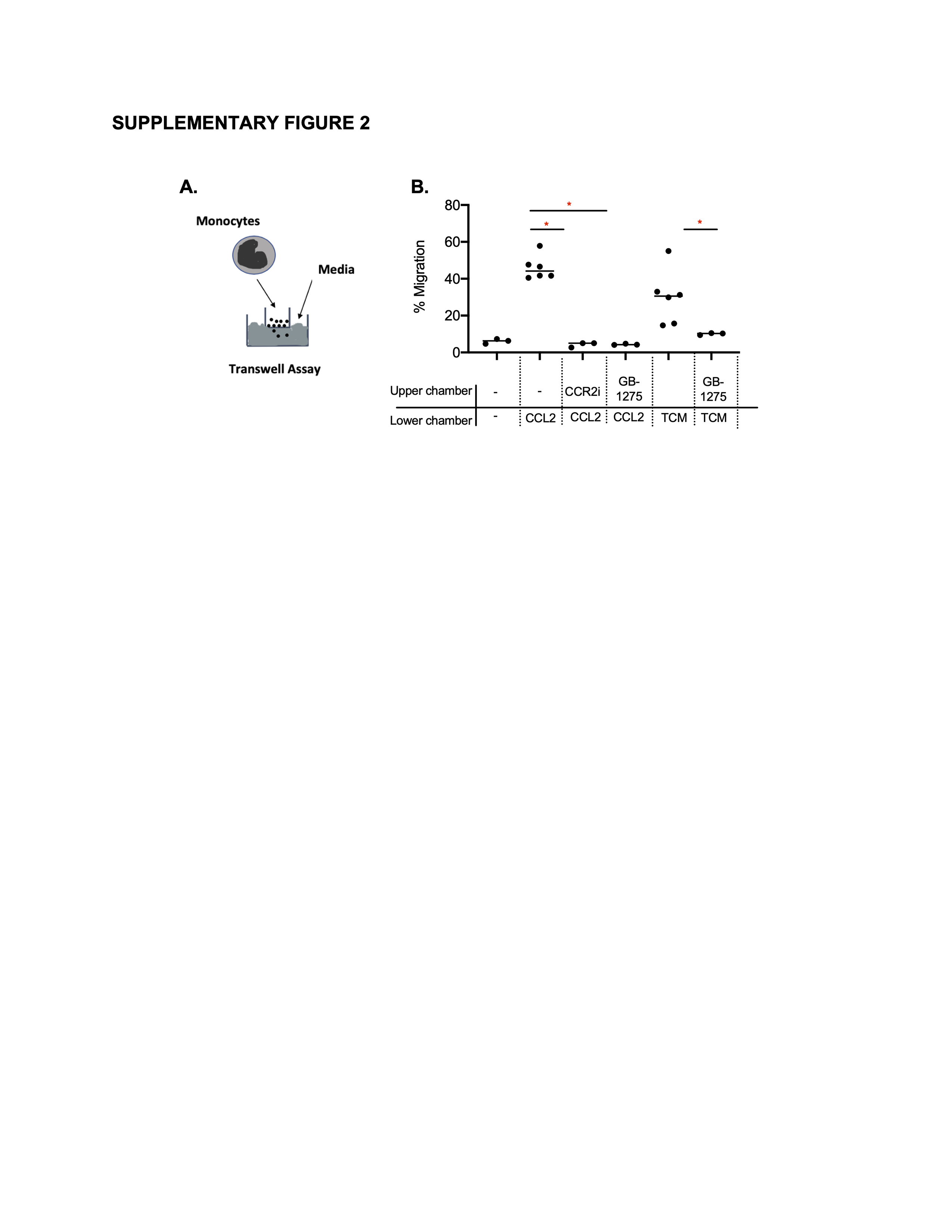

Supplement: Supplementary Figure 2 — (A) Schematic diagram describing the monocyte migration assay. (B) Migration assay analysis of THP1 human monocytes treated with media (–), 20 nM CCR2i (PF-4136309) or 20 μM GB1275. Pre-treated or untreated THP1 monocytes (5 × 10^5^) were seeded in the upper chamber and then submerged in media (–), 50 ng/mL CCL2, or tumor conditioned media (TCM) reported as % migration = cells counted in bottom chamber out of total cells seeded in upper chamber. *P < 0.05. Statistical analysis by Mann-Whitney t-test. [file Image_2.TIFF]

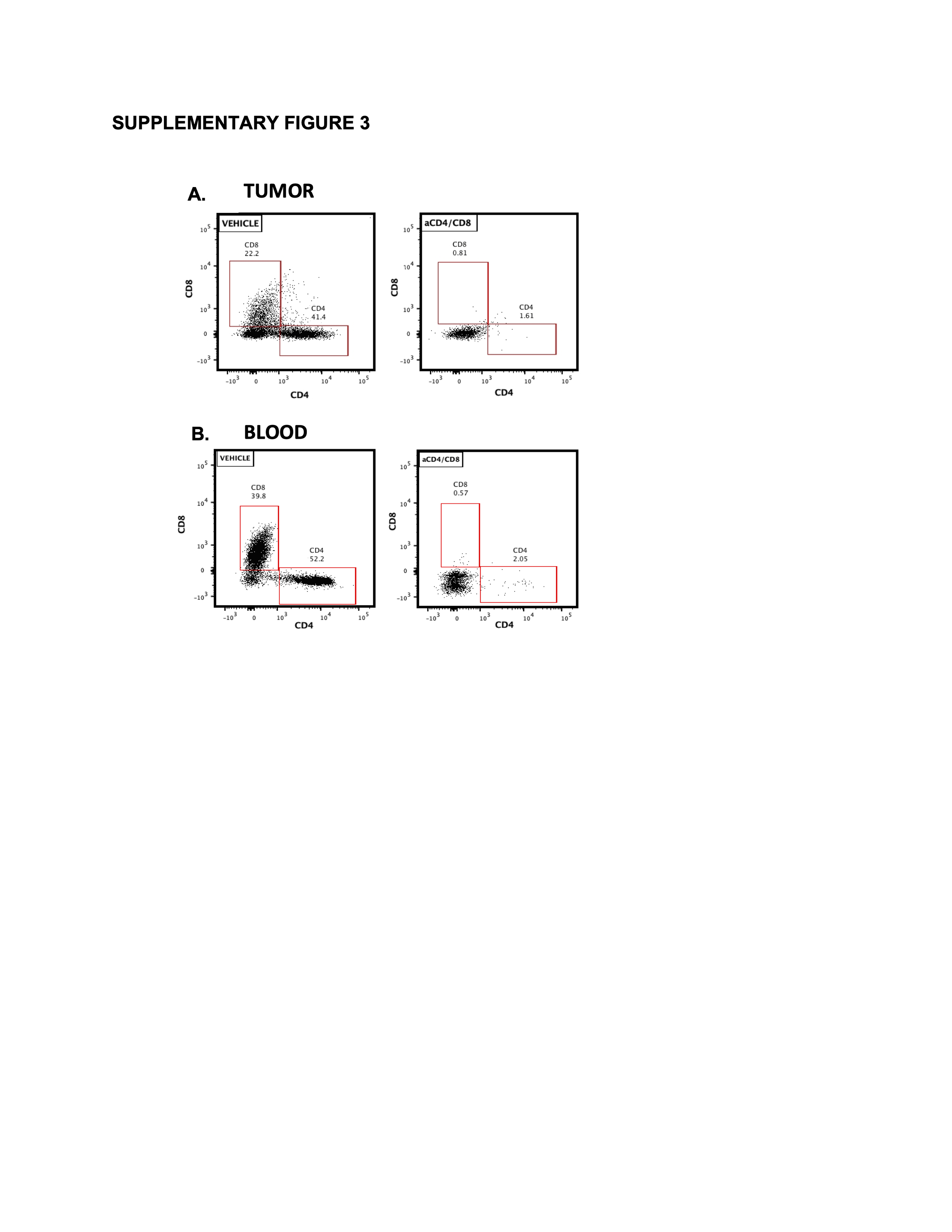

Supplement: Supplementary Figure 3 — Successful in vivo depletion of CD4 and CD8 T cell in LLC tumor-bearing mice. (A) Representative flow cytometry analysis of tumor cells from vehicle or anti-CD4/CD8 antibody depleted mice. (B) Representative flow cytometry analysis of peripheral blood cells from vehicle or anti-CD4/CD8 antibody depleted mice. Gated under live single cell, CD45+CD3+ cells. [file Image_3.TIFF]
