# Supplementary material for: Positive Allosteric Modulation of CD11b as a Novel Therapeutic Strategy Against Lung Cancer
Source: Front Oncol. 2020 May 21;10:748. doi: 10.3389/fonc.2020.00748 (PMC7253726; doi:10.3389/fonc.2020.00748)
Supplement: Supplementary Table 1 — Clinical characteristics of human lung adenocarcinoma tissues stained for CD11b in Figures 1A,B. Normal tissue acquiesced from the Northwestern University biorepository was either cadaveric or designated “non-tumor” normal tissue by pathology report. [file Table_1.DOCX]

| Patient # | 1 | 2 | 3 | 4 | 5 | 6 | 7 | 8 | 9 | 10 |
| --- | --- | --- | --- | --- | --- | --- | --- | --- | --- | --- |
| Gender | Female | Female | Male | Female | Female | Female | Male | Male | Male | Female |
| Age at Diagnosis | 62 | 76 | 70 | 57 | 59 | 53 | 69 | 49 | 73 | 64 |
| Age Last FollowUp | 63 | 78 | 72 | 58 | 63 | 54 | 70 | 56 | 73 | 69 |
| Smoking History | Never Assessed | Never Assessed | Former Smoker | Never Assessed | Former Smoker | Former Smoker | Former Smoker | Former Smoker | Former Smoker | Former Smoker |
| Age at Death | 63 | Alive | 72 | 58 | 63 | Alive | Alive | Alive | 73 | 69 |
| Family History | None | None | Father (bone cancer) | Mother (Breast) | Brother (site NA), Mother (site NA), Father (Prostate) | Maternal Uncle (Colon) | None | Maternal Aunt (Uterus) | None | Mother (Pancreatic), Father (Lung) |
| Race | White | White | White | White | Black | White | White | Black | White | White |
| Histology | Adeno-carcinoma | Adeno-carcinoma | Adeno-carcinoma | Adeno-carcinoma | Adeno-carcinoma | Adeno-carcinoma | Adeno-carcinoma | Adeno-carcinoma | Adeno-carcinoma | Adeno-carcinoma |
| Location | Left Lung | Left Upper Lobe | Left Lung | Left Lower Lobe | Right Lung | Left Lung | Left Upper Lobe | Left Lung | Left Upper Lobectomy | Right Middle Lobe |
| Differentiation | Moderate to Poor | Moderate | Moderate to Poor | Poor | Residual microscopic foci | Poor | NA | Moderate | Moderate | Poor |
| Size (cm) | 8 | 5.2 | 8 | 4.3 | 8.5 | 7.1 | 4.1 | 10 | 4.8 | 5.2 |
| T (Grade) | 3 | 3 | 2 | 2 | 1a | 3 | 2a | 4 | 2a | 2b |
| N (Node) | 1 | 2 | 2 | 2 | 1 | 2 | 2 | 1 | 2 | 2 |

**SUPPLEMENTARY TABLE 1**
