# Supplementary material for: Positive Allosteric Modulation of CD11b as a Novel Therapeutic Strategy Against Lung Cancer
Source: Front Oncol. 2020 May 21;10:748. doi: 10.3389/fonc.2020.00748 (PMC7253726; doi:10.3389/fonc.2020.00748)
Supplement: Supplementary Table 2 — List of antibodies used for flow cytometry analysis. [file Table_2.DOCX]

| Antigen | Clone | Fluorophore | Source |
| --- | --- | --- | --- |
| CD45 | 30-F11 | PE, APC/Cy7 | BioLegend |
| CD3 | 145-2C11 | BV421 | BioLegend |
| CD19 | 6D5 | BV421 | BioLegend |
| CD11b | M1/70 | AF488, AF700 | BioLegend |
| MHCII (I-A/I-E) | M5/114.15.2 | PE/Cy7 | BioLegend |
| Ly6G | 1A8 | AF488 | BioLegend |
| Ly6C | HK1.4 | BV605 | BioLegend |
| F4/80 | BM8 | APC | BioLegend |
| CD206 | C068C2 | PerCp/Cy5.5 | BioLegend |
| CD8a | 53-6.7 | AF700 | BioLegend |
| CD4 | RM4-5 | PE/Cy7 | BioLegend |
| CD11c | N418 | BV785 | BioLegend |
| CD24 | M1/69 | AF488 | BioLegend |
| CD103 | 2E7 | BV421, BV711 | BioLegend |
| Ki67 | 16A8 | PerCP/Cy5.5 | BioLegend |
| FoxP3 | FJK-16s | APC | BioLegend |
| Live/Dead Stain | N/A | Aqua -405nm | ThermoFisherScientific |

**SUPPLEMENTARY TABLE 2**
